# Impact of Upadacitinib on Atopic Keratoconjunctivitis Exacerbated by Dupilumab Treatment in Atopic Dermatitis Patients: A Prospective Dermatological and Ophthalmological Clinical Evaluation in Common Clinical Practice

**DOI:** 10.3390/jcm13133818

**Published:** 2024-06-28

**Authors:** Claudia Paganini, Sara Spelta, Lorenzo Tofani, Marina Talamonti, Luca Bianchi, Marco Coassin, Antonio Di Zazzo, Marco Galluzzo

**Affiliations:** 1 Department of Systems Medicine, University of Rome “Tor Vergata”, 00133 Rome, Italy; cld.paganini@gmail.com (C.P.); lorenzotofani1993@gmail.com (L.T.); luca.bianchi@uniroma2.it (L.B.); 2 Ophthalmology Complex Operative Unit, University Campus Bio-Medico, 00128 Rome, Italy; s.spelta@unicampus.it (S.S.); m.coassin@policlinicocampus.it (M.C.); a.dizazzo@policlinicocampus.it (A.D.Z.); 3 Dermatology Unit, Fondazione Policlinico “Tor Vergata”, 00133 Rome, Italy; talamonti.marina@gmail.com

**Keywords:** conjunctivitis, dupilumab, atopic keratoconjunctivitis, AKC, upadacitinib, DAOSD, atopic dermatitis

## Abstract

**Introduction:** Atopic dermatitis (AD) is a prevalent chronic inflammatory skin condition with a substantial impact on patients, particularly due to ocular involvement known as atopic keratoconjunctivitis (AKC). Current therapeutic approaches, such as dupilumab, often lead to conjunctivitis, prompting exploration of alternative treatments like upadacitinib. **Methods:** We collected dermatological and ophthalmological prospective clinical evaluations of six adults with moderate-to-severe AD, undergoing treatment with upadacitinib after discontinuation of dupilumab due to the onset of AKC during therapy and the worsening of dermatitis in particular in the head and neck region. Clinical evaluations, including EASI scores, itch and sleep NRS, DLQI, and ocular parameters, were performed at baseline (during screening assessment before switching to upadacitinib) and then at week 12 and week 24. Clinical evaluation of AKC was performed by a team of ophthalmologists. **Results**: Upadacitinib not only improved atopic dermatitis in terms of EASI, itching, and sleep NRS, but also demonstrated a notable reduction in ocular signs and symptoms, as indicated by the Visual Analogue Scale (VAS), the Efron scale, and the Ocular Surface Disease Index Symptom Severity (OSDISS) scores. **Discussion**: Our observation of common clinical practice underscores the substantial impact of biological and small-molecule therapies on AD, emphasizing the limitation posed by dupilumab-associated conjunctivitis. Switching to upadacitinib significantly improved both clinical and functional ocular outcomes, suggesting its potential as an alternative therapeutic option for AD patients with ocular involvement. **Conclusion:** The presented data provides insights into the complex interplay between systemic therapies and ocular manifestations in AD. Upadacitinib emerges as a promising option to address dupilumab-associated conjunctivitis, offering improved quality of life for patients.

## 1. Introduction

Atopic dermatitis (AD) is a common, chronic, relapsing, inflammatory skin disease characterized by severe itching, eczema, chronic lesions, and flares. In Europe and the USA, recent statistics indicate that around 20% of children have AD, while among adults, the prevalence varies between 7% and 14%, with significant differences observed between different countries [1].

Around 75% with childhood onset of the disease have a spontaneous remission before adolescence, whereas the remaining 25% continue to have eczema into adulthood or experience a relapse of symptoms after some symptom-free years. Two main hypotheses have been proposed to explain the inflammatory lesions in AD. The first hypothesis concerns an imbalance of the adaptive immune system (imbalance of T cells, particularly T helper cell types 1, 2, 17, and 22 and also regulatory T cells with Th2 differentiation of naive CD4+ T cells predominates); the second hypothesis concerns a defective skin barrier (mutations in the filaggrin gene; with gene defects, less filaggrin is produced, leading to skin barrier dysfunction and transepidermal water loss, which causes eczema) [2]. The most widely used diagnostic criteria for AD were developed by Hanifin and Rajka in 1980 and were later revised by the American Academy of Dermatology [3,4,5,6,7,8,9].

The most common ocular involvement in patients with AD is known as atopic keratoconjunctivitis (AKC) [4].

Ocular manifestations such as recurrent conjunctivitis, keratoconus, anterior subcapsular cataract, Dennie-Morgan infraorbital fold, and orbital darkening are considered minor criteria for diagnosis of AD according to the Hanifin-Rajka Diagnostic Criteria for AD [10].

Allergic conjunctival diseases (ACD) are a heterogeneous group of ocular diseases including allergic conjunctivitis [seasonal allergic conjunctivitis (SAC), perennial allergic conjunctivitis (PAC)] and immune-mediated conjunctivitis [giant papillary conjunctivitis (GPC, related to contact lenses use), AKC, and Vernal keratoconjunctivitis (VKC)]. The last two show a corneal involvement, in addition to conjunctivitis, and may develop moderate-to-severe ocular complications such as conjunctival scarring or corneal neovascularization, scarring, or erosions/ulcers; the Th2 pathway is thought to be responsible for its pathophysiology. Particularly, AKC is the ocular atopic disease related to AD, while Vernal keratoconjunctivitis develops more commonly in young males during the springtime and in hot and dry environments [11,12,13,14].

VKC is clinically characterized by bilateral bulbar conjunctival injection with associated watery and mucoid discharge. Patients develop a giant papillary hypertrophy of only the superior tarsal conjunctiva, resembling “cobblestones”. Specific findings that help differentiate VKC from AKC are limbal (Horner-Trantas) dots which are small white-yellow chalky concretions around the corneal limbus; corneal vernal plaques; or shield (Togby’s)-shaped ulcers of the cornea [15].

**Figure 1:**
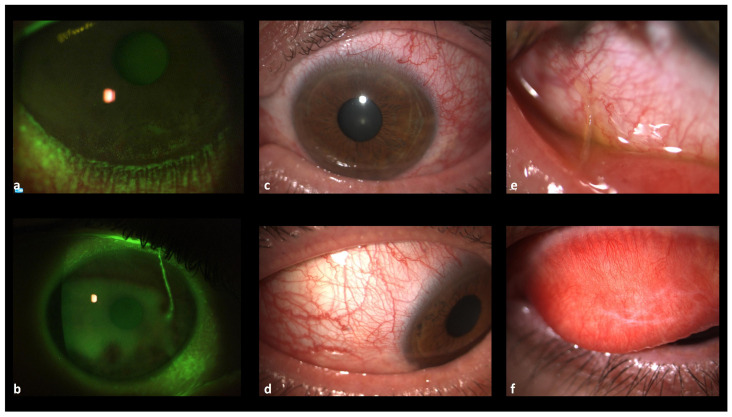
(**a**). Superficial punctate keratitis in the inferior cornea and reduced BUT (tear break-up time). (**b**). Mucous filaments highlighted by fluorescein staining. (**c**). Conjunctival hyperemia with corneal neovascularization in the upper limbus and Meibomian gland dysfunction. (**d**). Upper eyelid tarsal papillary reaction, more evident with fluorescein staining. (**e**). Mucous filaments, conjunctival hyperemia and increased tear meniscus. (**f**). Long-term tarsal fibrosis with hyperemia and papillary reaction.

AKC clinical signs include papillary hypertrophy, conjunctival injection, mucous filaments, meibomian glands dysfunction and eczematoid blepharitis, mucocutaneous junction involvement up to trichiasis, punctate superficial keratitis, and eyelid thickening with subepithelial conjunctival fibrosis and scarring; in more severe and untreated cases, patients can develop loss of eyelashes, symblefaron, corneal neovascularization and keratinization, corneal ulcers or scars, punctate epithelial keratitis and associated keratoconus, and/or anterior/posterior subcapsular cataracts [16] (Figure 1).

AKC may cause blindness through corneal neovascularization and opacities, as well as destruction of corneal epithelial stem cells and cicatricial sequelae [17]. However, despite strict therapy, such patients experience a critical reduction in their quality of life. Ocular discomfort, itching, and visual impairment limit their daily activities (driving, working, meeting friends) as well as their personal, social, and psychological development in this young patient group.

AKC develops in 25–40% of AD patients [7,11,18] and is more common in AD patients with head–neck involvement [19].

Standard treatment of AKC includes lubricating eye drops, topical antihistaminic and mast-cell stabilizers, topical steroids, and topical cyclosporine [20].

Keratoconus is considered one of the ocular complications of AKC [21,22]. Eye-rubbing has been implicated in the pathogenesis of keratoconus [23]. Eye rubbing seems to cause the thinning of keratocyte, and the degree of effect of eye rubbing depends on the period and force of the performed eye rubbing [24,25]. Keratoconus is significantly more common in patients with AKC and VKC compared to the normal population. It is associated with faster progression and a more severe course of disease [26,27].

New systemic drugs for the treatment of AD include anti-interleukins and JAK inhibitors [28,29,30,31].

Dupilumab is an anti-interleukin IL-4Ra antibody that inhibits IL-4 and IL-13 and secondary intracellular pathway. Upadacitinib is a novel selective inhibitor of Janus kinase 1 approved for AD patients [32,33,34].

The main RCT for the commercialization of dupilumab SOLO 1 and 2, CHRONOS and CAFE, reported a higher incidence of conjunctivitis in patients receiving dupilumab for AD compared to those receiving a placebo (11%) [35]. In addition, the same trials reported a higher incidence of conjunctivitis in patients receiving dupilumab for AD compared to those receiving dupilumab for asthma/nasal polyposis (20% vs. 1%). This suggests that factors unique to AD may play a role in its development [9,36].

A meta-analysis from 2021 involving 3303 patients highlighted that 26.1% of the patients developed conjunctivitis during the therapy. Dupilumab appears to exacerbate ocular symptoms and signs such as conjunctival redness, papillary reaction, and superficial punctate keratitis. Dupilumab-associated ocular surface disease (DAOSD) seems to occur more frequently in the early months of treatment, with a disease severity ranging from mild to moderate and is more common in patients with involvement of the head and neck region [19,36,37,38,39,40,41].

Several mechanisms have been proposed in the etiopathogenesis of DAOSD: pre-existing subclinical inflammatory processes of atopy or inflammatory allergic diseases; qualitative dysfunction of tear film; local deficit of immune system with increased susceptibility to bacterial, viral, chlamydia, and mycoplasma infection; increased expression of pro-inflammatory molecules (e.g., OX40L) secondary to immune microenvironment alteration; Demodex spp. colonization of Meibomian glands; hyper-eosinophilia; IL-13 reduction leading to conjunctival goblet cell impairment; immune-mediated responses by conjunctival-associated lymphoid tissue (CALT); and reduced dupilumab function in the ocular site due to the drug-increased washout and reduced bioavailability [37,42].

Another well-described phenomenon during dupilumab treatment is dupilumab facial redness (DFR) or the development of an eczematous facial rash. It occurs in approximately 4–43.8% of patients on dupilumab and may be seen in adults as well as children. There has been speculation as to whether DFR has an association with DAOSD, but there is currently no well-established association between these two adverse events [43].

Recent studies have shown improvement in DAOSD by switching to upadacitinib therapy [41,44]. It is not yet clear whether dupilumab treatment is responsible for the onset or exacerbation of allergic conjunctivitis.

Upadacitinib has been recently introduced for the treatment of severe AD without reporting ocular adverse events in the registration studies [35]. As of now, the prescription of upadacitinib in common clinical practice as a first-line treatment disregards the absence of risk factors indicated by the EMA (age equal to or greater than 65 years, increased risk of serious cardiovascular problems, long-term smokers or ex-smokers, or at higher risk of cancer), and is used solely upon the failure (inefficacy/loss of efficacy, occurrence of adverse events, contraindications to treatment) of cyclosporine.

We collected dermatological and ophthalmological prospective clinical evaluations of six adults with moderate-to-severe AD, undergoing treatment with upadacitinib after discontinuation of dupilumab due to the onset of AKC during therapy and worsening of dermatitis, in particular in the head and neck region. Clinical evaluation of AKC was performed by a team of ophthalmologists.

## 2. Materials and Methods

### 2.1. Study Design

Our observation is only a collection of dermatological and ophthalmological prospective clinical evaluations in common clinical practice of six adults with moderate-to-severe AD, undergoing treatment with upadacitinib after discontinuation of dupilumab due to the onset of AKC during therapy and worsening of dermatitis, particularly in the head and neck region. Every patient was observed for at least 6 months. Each clinical evaluation was part of the activities of common clinical practice (without any type of instrumental or therapeutic intervention alternative to what is normally performed in common clinical practice). As suggested in the literature, an ophthalmological assessment in patients affected by AD with signs of AKC is part of the common clinical activity.

The main aim of this study was to evaluate the severity of AKC induced by dupilumab and to assess any improvement in the ocular condition by discontinuing dupilumab and treating the same patients with upadacitinib.

The research involved adult individuals aged 18 years and older who were undergoing single-drug therapy at the Dermatology Unit of the Tor Vergata Polyclinic Foundation in Rome, Italy. These patients also underwent eye examinations at the Ophthalmological Unit of the Campus Biomedico Foundation, also located in Rome, Italy. Information was gathered between April 2023 and January 2024.

**Figure 2:**
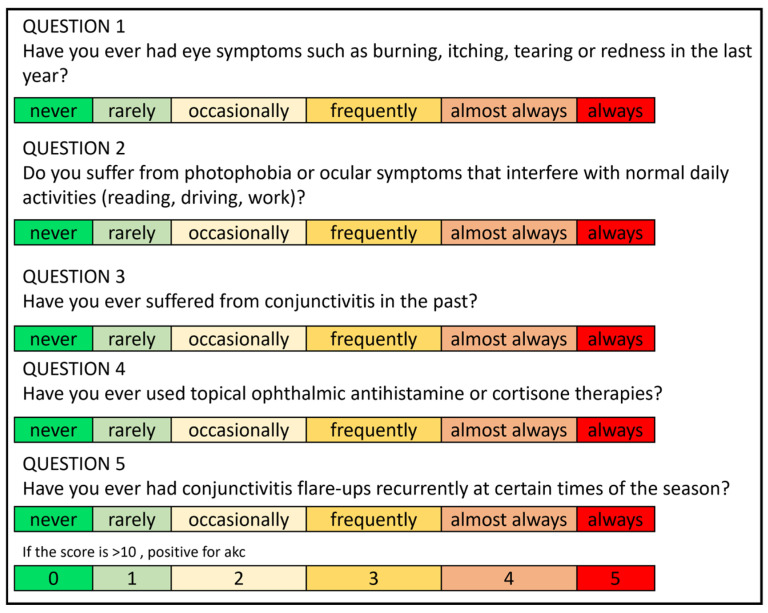
Scoring system adapted from Peter Foley et al. regarding ocular red flags. Each question is scored from 0 to 5 points. The score is considered positive for AKC when it exceeds 10 points [46].

The assumption of AKC was made using the scoring system based on a study regarding ocular red flags [45] (Figure 2).

In patients with a positive score for AKC diagnosis suspicion (the score is considered positive for AKC when it exceeds 10 points), and with poor control of the signs and symptoms of atopic dermatitis, dupilumab was discontinued. After a thorough screening and an 8-week washout period from dupilumab, patients were initiated on upadacitinib therapy.

At the initiation of upadacitinib treatment, patients underwent their first ophthalmologic assessment. Patients with a previous medical history of ocular and/or systemic inflammatory, autoimmune, autoinflammatory, allergic, congenital disease, or who had undergone prior ocular surgery or had any previous or concomitant ocular diseases (including glaucoma) were excluded. Participants were not currently using any topical or systemic anti-inflammatory or antiglaucoma drugs or treatments that might alter tear film secretion (e.g., beta-blockers, antidepressants, psychotropics). Participants had no history of ocular or peri-ocular malignancies or premalignant conditions, active or suspected ocular or peri-ocular infection and no allergy to dupilumab/upadacitinib. Patients were excluded if they were pregnant or planning pregnancy or if they received a previous diagnosis of severe AKC refractive to topical antihistamines.

Patients were chosen by examining their medical histories, a task undertaken by the participating physicians. The use of upadacitinib in patients with AD adhered to the guidelines outlined in the Summary of Product Characteristics (15 mg/day), encompassing individuals who exhibited either inadequate response, contraindications, or adverse reactions to at least one traditional systemic therapy as per Italian regulations.

At enrolment, age, sex, body mass index (BMI), age when AD started, duration of the disease, phenotype of AD, involvement of head and neck region, other health issues, and past and current treatments were recorded (Table 1).

The severity of the cutaneous disease was evaluated utilizing various metrics: (a) the Eczema Area Severity Index (EASI), graded on a scale of 0 to 72; (b) the Itch Numeric Rating Scale (itch-NRS), with ratings ranging from 0 to 10; and (c) the Sleeplessness Numeric Rating Scale (sleep-NRS), scored between 0 and 10.

The ocular parameters considered were mucous filaments, conjunctival hyperemia, corneal neovascularization, corneal opacities, papillary reaction, tarsal fibrosis, tear breakup time (BUT), and corneal staining with fluorescein.

Prior to enrollment, all patients provided written consent, and this study adhered to the ethical principles delineated in the 1975 Declaration of Helsinki. It is noteworthy that, in accordance with Italian regulations, studies of this nature do not necessitate formal approval from an ethics committee [47].

The effectiveness of upadacitinib therapy was assessed by quantifying the decrease in EASI, itch NRS, and sleep NRS scores at both week 12 and week 24, along with the simultaneous reduction in ocular manifestations of AKC.

### 2.2. Study Population

The study cohort comprised six adult patients aged 18 years and older. The average age of the participants was 34.30 years (SD 14.94), with four of them being male. The mean duration of the disease was 25.16 years (SD 19.37), and the average duration of dupilumab treatment was 24.6 months (SD 13.89). All patients exhibited involvement of the head–neck region. At the beginning of dupilumab treatment, the mean Eczema Area Severity Index (EASI) score was 35.33 (SD 10.61), whereas, at the onset of upadacitinib treatment, the mean EASI score was 20.17 (SD 7.62).

### 2.3. Outcome Measures

Screening ophthalmic visit (W0) was conducted prior to starting systemic therapy with upadacitinib. At all study visits, patients completed the VAS, Esprint-15, DECA, and rTOSS questionnaire and underwent slit lamp clinical examination [48,49]. Data were collected concerning Corneal Vital Staining Score (National Eye Institute [NEI] score 0–15), tear break-up time (TBUT), meibomian gland dysfunction (MGD), the severity of conjunctival hyperemia (assessed through Efron scale), and ocular complications (OSDISS score) at screening and at following visits.

All patients were given the standard treatment for AD, oral upadacitinib 15 mg, as it was the only reimbursable dosage in Italy at the time of observation.

For dermatological visits, baseline assessments were conducted, followed by visits at week 12 and at week 24. During each visit, evaluations were made for the absolute EASI score, DLQI, NRS itching, and NRS sleep. Given that patients in this observation initiated treatment at different time points, the presented data should be regarded as a snapshot, offering a cross-sectional representation of our experience until January 2024.

### 2.4. Statistical Analysis

Continuous variables are reported as mean ± standard deviation (SD) and categorical variables as number and percentage. The analysis was conducted using the intention-to-treat (ITT) population. All statistical surveys were performed using SPSS (IBM) software and RStudio open-source statistical software. Comparisons between average scores used the analysis of variance (ANOVA) associated with an internal post hoc analysis (Dunnett’s, Tukey Kramer, Bonferroni) after a normality check (Shapiro test). *p* values < 0.05 were considered statistically significant (95% confidence value). The McNemar test was used for the analysis of qualitative variables. A power of 80% was considered sufficient to detect statistically significant differences at an alpha level of 0.05 with a large, standardized difference between the four groups (balanced one-way analysis of variance power calculation: k = 4, f = 0.4, sig. level = 0.05, power = 0.8; k = 4, n = 19.10, f = 0.4, sig. level = 0.05, power = 0.8).

## 3. Results

Data were analyzed for six patients who had been treated with dupilumab for at least 24 weeks and then switched to upadacitinib due to AKC onset and loss of efficacy in controlling cutaneous disease, particularly in the head–neck region. Upadacitinib demonstrated significant efficacy in substantially reducing multiple outcome measures, both dermatological and ophthalmological, at each designated assessment time point (week 12 and week 24) compared to baseline measurements.

The average EASI score exhibited a significant decrease from a baseline of 20.17 (SD 7.62) to 2.0 (SD 3.34) at week 12, maintaining this improvement at week 24 with a mean score of 1.6 (SD 2.06) (*p* < 0.001).

Similarly, the mean itch Numeric Rating Scale (itch-NRS) score, a key indicator of symptom severity, decreased from 8.83 (SD 1.33) to 2.16 (SD 1.94) over the 24-week duration. This highlights a substantial alleviation of itching symptoms, evident as early as week 12, with an average score of 2.66 (SD 3.26) (*p* < 0.01).

**Figure 3:**
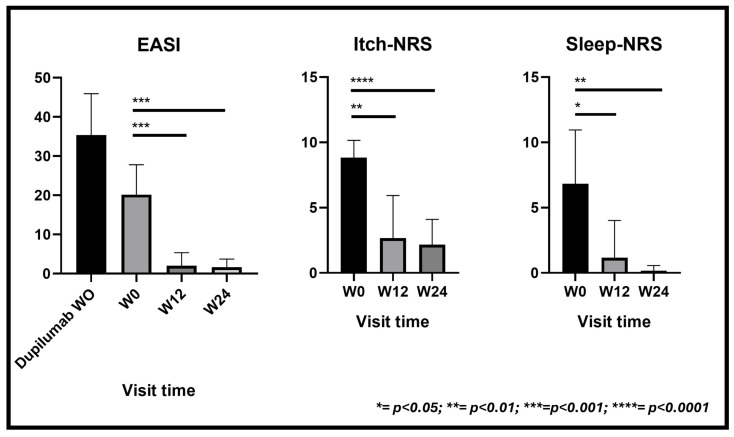
Effect of upadacitinib on EASI score and itch and sleep NRS over 24 weeks in patients with AD. Missing values for intermediate visits were imputed with the last observation carried forward (LOCF) method. Dupilumab W0 means mean EASI before initiating dupilumab; W0 means mean EASI after discontinuing dupilumab, before initiating upadacitinib.

The quality of sleep, assessed through the mean sleep-NRS score, exhibited enhancement from an initial value of 6.83 (SD 4.11) to 0.16 (SD 0.40) by week 24 (*p* < 0.05) (Figure 3).

The main ocular symptoms evaluated include itching/burning, tearing, and redness individually (rTOSS questionnaire) and collectively (VAS score), the impact of these symptoms on daily life activities (DECA criteria), and the presence of ocular itching in the last 2 weeks (question 15 of the Esprint questionnaire) [48,49].

**Figure 4:**
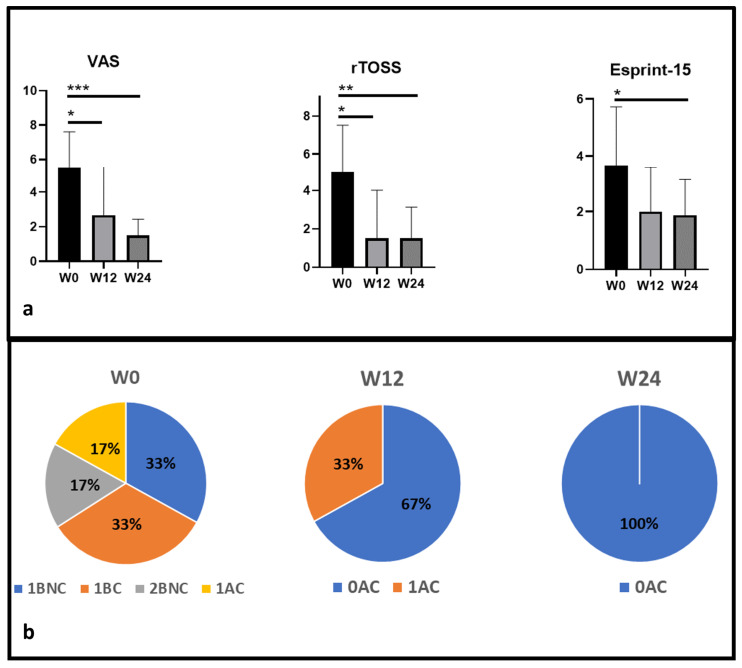
(**a**): Effect of upadacitinib on ocular symptoms. VAS, rTOSS, and Esprint-15 show statistically significant reduction from W0 to W24. (**b**): Effect of upadacitinib on impact of ocular symptoms on quality of life, according to DECA criteria (0 = mild, 1 = moderate, 2 = severe, A = intermittent, B = persistent, C = controlled, NC = not controlled). At W0, 17% of patients presented 1AC score, 33% 1BC, 33% 1BNC, and 17% 2BNC, while at W24, all patients reached the 0AC score, suggesting great improvement in patients’ symptoms and in daily life activities. * = *p* < 0.05; ** = *p* < 0.01; *** = *p* < 0.001.

Results indicate a statistically significant reduction in all scores (VAS, rTOSS, Esprint-15, and DECA criteria) over time from week 0 to week 24 (Figure 4).

**Figure 5:**
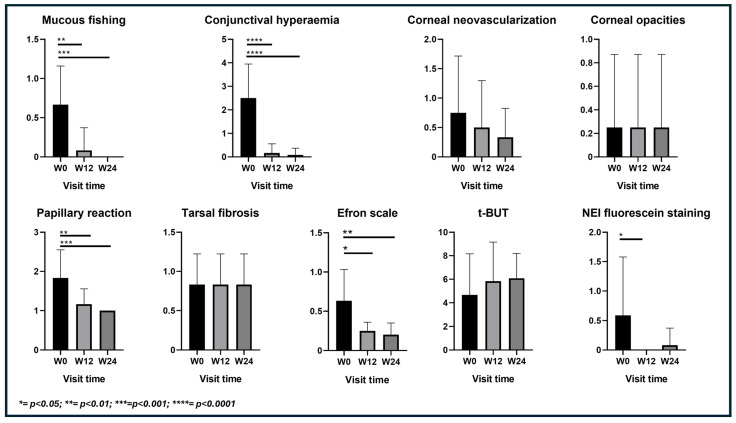
Effect of upadacitinib on ocular signs. Mucous fishing, conjunctival hyperemia, papillary reaction, and NEI corneal staining reached a statistically significant reduction at 3 months and 6 months (except for NEI) from starting upadacitinib. Corneal neovascularization shows a decreasing trend, while BUT highlights an increasing trend. A minimal effect of therapy exists on the tarsal fibrosis, papillary reaction, and corneal opacities, since there are not reversible signs.

The treatment with upadacitinib significantly reduces the clinical signs of ocular inflammation (mucus filaments, conjunctival hyperemia, papillary reaction, and NEI) in a statistically significant manner between the time points week 0, week 12 and week 24 (except for NEI which is only between W0 and W12), while it does not have an effect (at least in the short term) on irreversible signs (subconjunctival tarsal fibrosis and corneal opacities) (Figure 5).

In particular, the mean mucous fishing showed a significant reduction from 0.66 (SD 0.50) at baseline to 0.08 (SD 0.28) at week 12, maintaining this value at week 24 with a mean score of 0.00 (SD 0.00) (*p* < 0.001). Similarly, the average values of conjunctival hyperemia, decreased from 2.50 (SD 1.44) to 0.08 (SD 0.28) over the 24-week period (*p* < 0.0001). This underscores a notable amelioration of ocular inflammation. Furthermore, the mean papillary reaction showed a decrease from 1.83 (SD 0.71) to 1.00 (SD 0.00) over the 24-week period.

The average values of the Efron scale show a reduction over time. Meibomian gland dysfunction (MGD) and tear break-up time (BUT) values are concordant and inversely proportional; as MGD decreases, BUT increases, indicating an improvement in tear film quality from week 0 to week 12.

The corneal neovascularization shows a decreasing trend (which is positive because it indicates that chronic inflammation is decreasing over time), but there is a limitation in statistical significance due to the small sample size of patients. The BUT shows an increasing trend, indicating an improvement in the functionality of the Meibomian glands and the quality of the tear film, although the data is not statistically significant.

All the ocular parameters are summarized in Table 2.

### 3.1. Clinical Cases

#### 3.1.1. Case 1

A 25-year-old man with a medical history including asthma, rhinitis, and conjunctivitis has been grappling with AD since the age of 5. Despite prior treatments involving topical corticosteroids and antihistamines yielding minimal benefits, he underwent biological therapy with dupilumab at another specialized center. Initially, he experienced improvements during the first 4 months of treatment, but subsequently faced diminishing efficacy, particularly in the head and neck area, leading to the decision to discontinue dupilumab.

In March 2023, he presented with severe AD predominantly affecting the head and neck region, alongside significant ocular discomfort. The EASI was assessed at 15, and the itch NRS score, sleep NRS score, and DLQI were 7, 10, and 20, respectively. Ophthalmological evaluation revealed conjunctival hyperemia, tarsal fibrosis, papillary reaction, and dysfunction of Meibomian glands with altered tear film break-up time (BUT), culminating in a diagnosis of AKC.

**Figure 6:**
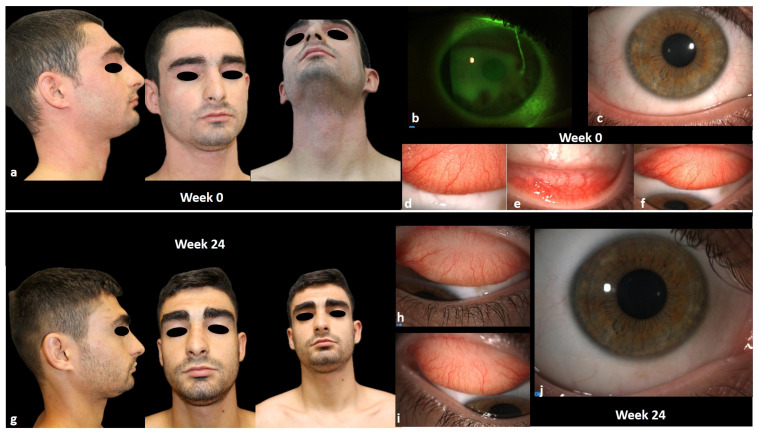
(**a**–**f**): ocular signs (conjunctival hyperemia, papillary reaction, mucous fishing, NEI score, Efron scale) and head and neck AD at baseline; (**g**–**j**): ocular signs (conjunctival hyperemia, papillary reaction, mucous fishing, NEI score, Efron scale) and head and neck AD at week 24.

In May 2023, after the analyses which also included the evaluation of hepatitis, the TB gold quantiferon, a chest X-ray, and a cardiological examination, upadacitinib was administered at a dose of 15 mg daily. An excellent response was observed in the first 6 weeks of treatment, characterized by significant improvement in disease parameters such as EASI, itch and sleep NRS scores, and a notable enhancement in quality of life. Ophthalmic pictures highlighted the great improvement in patient’ symptoms (itching, burning, tearing, foreign body sensation) and clinical signs (absence of conjunctival hyperemia and mucous filaments, increased BUT with better quality of tear film and less MGD) (Figure 6).

The patient is currently on 10 months of continuous treatment with upadacitinib 15 mg, and he is still in complete remission (both EASI, itch and sleep NRS, and DLQI equal to 0).

#### 3.1.2. Case 2

A 19-year-old man with a history of allergic rhinitis has been affected by AD since the age of 15. He was previously treated with topical corticosteroids and antihistamines with little benefit. He underwent treatment with dupilumab from January 2020 to May 2023, at which point the drug was discontinued due to secondary ineffectiveness in the head and neck region and the onset of AKC. During dupilumab therapy, the patient initiated lubricating eyedrops and topical tacrolimus/steroids to treat ocular symptoms, with incomplete benefit.

In July 2023, after accurate instrumental and laboratory screening tests, upadacitinib was administered at a dose of 15 mg daily.

He presented with severe AD, primarily affecting the head and neck region, and complained of significant ocular discomfort. Evaluation of the EASI was 15, and the itch NRS score, sleep NRS score, and DLQI were 10, 10, and 20, respectively. The ophthalmological evaluation revealed chronic inflammation of the ocular surface due to the AKC: severe conjunctival hyperemia and limbitis, mucous filaments, papillary reaction with tarsal fibrosis, superficial punctate keratitis, and Meibomian glands dysfunction with reduced BUT.

An excellent response was observed in the first 6 weeks of treatment, characterized by significant improvement in disease parameters such as EASI, itch and sleep NRS scores, and a notable enhancement in quality of life. Ophthalmic evaluation showed a remarkable improvement in ocular inflammation (normoemic conjunctiva without mucous filaments, superficial punctate keratitis resolved, improvement in BUT) and in the patient’s symptoms (itching, burning, tearing, foreign body sensation).

**Figure 7:**
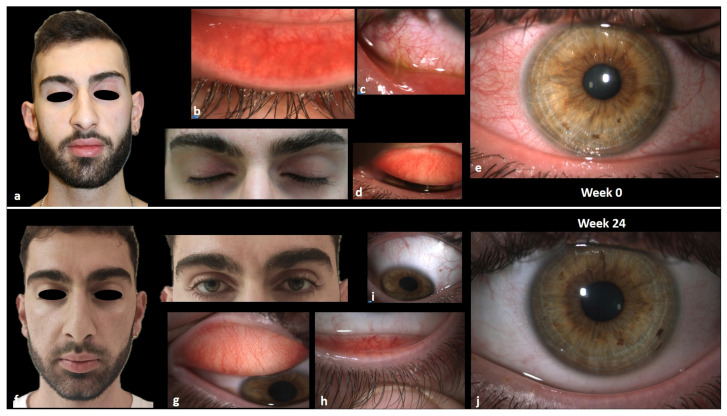
(**a**–**e**): ocular signs (conjunctival hyperemia, papillary reaction, mucous fishing, NEI score, Efron scale) and head and neck AD at baseline; (**f**–**j**): ocular signs (conjunctival hyperemia, papillary reaction, mucous fishing, NEI score, Efron scale) and head and neck AD at week 24.

The patient is currently on 8 months of continuous treatment with upadacitinib 15 mg, and he is still in complete remission (both EASI, itch and sleep NRS, and DLQI equal to 0) (Figure 7).

#### 3.1.3. Case 3

A 24-year-old man with a history of allergic rhinitis has been affected by AD since the age of 16. He was previously treated with topical corticosteroids and antihistamines with little benefit.

He presented in May 2020 with a severe AD. Evaluation of the EASI was 40, and the itch NRS score, sleep NRS score, and DLQI were 10, 8, and 5, respectively. In June 2020, dupilumab was started. An excellent response was obtained in the first 16 weeks of treatment, highlighted by an almost clearing of disease parameters such as EASI and NRS itch and sleep score and by a significant improvement in the quality of life. However, he was monitored by his ophthalmologists for ocular symptoms and signs since his childhood, and he was treated with topical steroids and antihistamines for a long time. In addition, he developed bilateral and progressive keratoconus. Therefore, he underwent corneal crosslinking when he was 23 years old.

Unfortunately, after three years of treatment with dupilumab, in May 2023, due to a loss of efficacy in the head and neck region and concurrent conjunctivitis, we decided to discontinue dupilumab.

In July 2023, after the analyses which also included the evaluation of hepatitis, the TB gold quantiferon, a chest X-ray, and a cardiological examination, upadacitinib was administered at a dose of 15 mg daily.

Evaluation of the EASI was 15, and the itch NRS score, sleep NRS score, and DLQI were 10, 4, and 15, respectively. The ophthalmological evaluation discovered the chronic inflammation of the ocular surface secondary to the AKC, severe conjunctival hyperemia and limbitis, corneal neovascularization, mucous filaments, papillary reaction with tarsal fibrosis, increased tear meniscus, and Meibomian glands dysfunction with reduced BUT.

**Figure 8:**
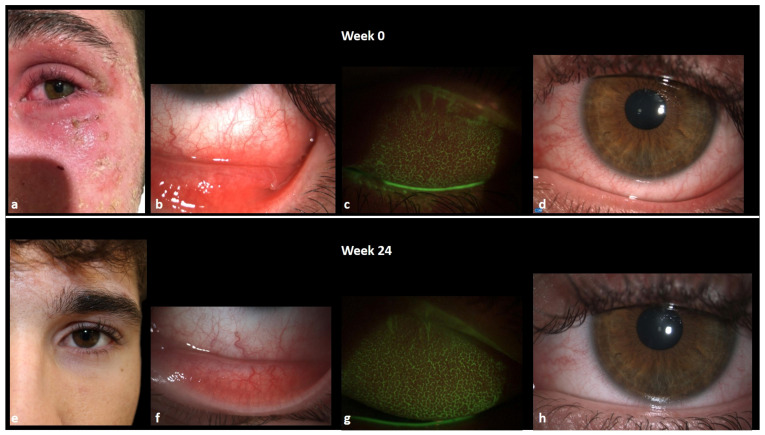
(**a**–**d**): ocular signs (conjunctival hyperemia, papillary reaction, mucous fishing, NEI score, Efron scale) and head and neck AD at baseline; (**e**–**h**): ocular signs (conjunctival hyperemia, papillary reaction, mucous fishing, NEI score, Efron scale) and head and neck AD at week 24.

An excellent response was observed in the first 12 weeks of treatment, characterized by significant improvement in disease parameters such as EASI, itch and sleep NRS scores, and a notable enhancement in quality of life. Ophthalmic evaluation revealed a great improvement in ocular inflammation (normoemic conjunctiva without mucous filaments, resolved corneal neovascularization, reduced tear meniscus, improvement in BUT) and in the patient’s symptoms (itching, burning, tearing, foreign body sensation) (Figure 8).

The patient is currently on 9 months of continuous treatment with upadacitinib 15 mg, and he is still in complete remission (both EASI, itch and sleep NRS and DLQI equal to 0).

## 4. Discussion

The impact of biological drugs and small molecules on the treatment of AD is significant, markedly reducing dermatological signs and symptoms, as demonstrated by EASI scores, DLQI, and the itch and sleep NRS. However, a major limitation of using dupilumab in AD patients is the onset of conjunctivitis, which significantly reduces the quality of life for these patients. These individuals are mostly young, primarily adolescents, who psychologically suffer from both the worsening of body image due to AD and eye-related issues. This affects the quality of their vision, impacting daily activities, especially studying and working, and their self-perception, with a strong impact on social and relational life. Our observations of common clinical practice revealed that switching to upadacitinib therapy significantly improved not only atopic dermatitis not sufficiently controlled with dupilumab, but also clinical ocular signs (demonstrated with functional and anatomical outcome improvement) and ocular symptoms, as demonstrated by VAS, rTOSS, Esprint, and DECA scores. In fact, the latter indices measured after switching to upadacitinib suggest that symptoms have a significantly lower impact on daily activities, making patients feel more “free” from their disease if compared with the same indices measured during dupilumab treatment. Patient-reported subjectivity is further confirmed by the remarkable improvement in clinical signs, both dermatological and ophthalmic. In detail, the analysis showed a rapid decrease in conjunctival redness, papillary reaction, mucous discharge, Meibomian gland dysfunction, superficial punctate keratitis (NEI score), and scores grouping all clinical signs and complications (Efron scale and OSDISS score, respectively). Additionally, the improvement in Meibomian gland function is related to the increase in BUT, highlighting a better tear film quality, and a consequent reduction in the Schirmer test (the increase in tearing at baseline is secondary to hyper-evaporative dry eye due to tear film alteration, with a positive feedback mechanism on the lacrimal gland).

Considering the Heads Up registrational study (upadacitinib 30 mg vs. dupilumab 300 mg), which shows a significantly lower percentage of conjunctivitis in the upadacitinib group vs. the dupilumab group (1.4% vs. 10.2%, respectively), and different registrational studies of dupilumab in AD patients vs. patients with asthma and nasal polyposis, where the incidence of conjunctivitis in the AD group is considerably higher than the asthma/nasal polyposis group, our hypothesis is to consider the onset of DAOSD as AKC rather than a side effect of dupilumab [9].

In more detail, the literature data shows that allergic conjunctivitis occurring in a patient with AD is, by definition, AKC [46]. The diagnosis of AKC is often unrecognized by most ophthalmologists and, therefore, is often delayed or incorrect. Many AD patients report having suffered from ‘conjunctivitis’, ‘red eyes’, and ‘secretions’ since childhood but never received a diagnosis of AKC. Consequently, the initiation of dupilumab therapy could unmask or fail to control the underlying ocular disease, making AKC clinically apparent (already present but undiagnosed). In 1991, Foster et al. reported that an immunity mediated by T-cells was the cause of AKC and its chronicity [50].

IFNγ, which is increased in tears of patients with corneal damage, significantly correlated with the corneal score, suggesting that the over-production of this pro-inflammatory cytokine might be related to a worsening of the allergic inflammation. IFNγ is increased in chronic conjunctivitis, AKC, and VKC, and is a key cytokine in TH1 inflammation [51].

Other authors have confirmed that sparing IL-4 in the selective blockade of interleukin-13 in patients treated with tralokinumab leads to a smaller Th2/Th1 immune switch and a lower incidence of conjunctivitis compared to those treated with dupilumab [52,53]. In contrast, during dupilumab therapy, an AKC-like disease may arise due to the activation of alternative cytokine pathways, such as IL-33, resulting from the blockade of the IL-4/13 pathway. IL-33 is thought to play a significant role in atopic conditions, as recently suggested by Chiricozzi et al. [54].

Our hypothesis, which will be confirmed by molecular results, assumes a Th2 to Th1 switch induced by dupilumab, leading to a reactivation of underlying AKC. In contrast, upadacitinib, by blocking both Th2 and Th1 axes, could control both ocular symptoms and signs. We consider our observation a pilot study. Obviously, molecular and real-life studies are needed to support the hypothesis derived from this study. In this context, a recent publication confirms our assumption that AD itself is associated with underlying inflammation of the ocular surfaces. Blocking IL-13 activity with dupilumab leads to two major consequences: first, it inhibits the proliferation and function of goblet cells, resulting in decreased mucus production and barrier dysfunction, akin to that seen in dry eye disease; second, dupilumab induces a shift in the cytokine profile from a mixed Th2/Th17 to a Th1/Th17 pattern in AD patients who develop DAOSD. Both the reduction in goblet cell function and the cytokine shift may affect the type and intensity of inflammation, subsequent tissue remodeling, and the ocular surface microbiome [55].

In this sense, a more recent Canadian expert consensus suggests that when choosing a systemic treatment option for patients with a history of severe OSD, the treating dermatologist could consider starting with a JAKi or traditional systemic agent. If initiating an IL-4 and/or IL-13 inhibitor, consider an ophthalmology assessment prior to the commencement of treatment [56].

## 5. Conclusions

AKC is an ocular condition often found in patients with AD. Ophthalmic involvement should be studied and assessed by experienced allergo-immunology ophthalmologists to capture the early stages of the disease and block its progression at the outset.

Currently, the exact mechanism of AKC onset cannot be established, and there are no predictive or preventive indices available. Further studies will be necessary to understand the exact connection between dupilumab therapy and resistance in the head–neck region, which leads to the onset of ‘red face’ and DAOSD. For this reason, we emphasize the importance of an ophthalmological evaluation before starting any type of systemic therapy in patients suffering from AD. The prospects and challenges for the coming years will involve framing AD as an “atopic syndrome” with the creation of multidisciplinary teams (including ophthalmologists) and diagnostic therapeutic-assisted paths (DTAPs). This approach aims to ensure that patients are monitored for all Th2-mediated inflammation comorbidities without diagnostic delays or at least with reduced delays compared to current standards. Moreover, the dogma that atopy is a disease solely within the Th2 immune axis is gradually being overcome, opening up new avenues for research and the study of new therapeutic targets.

In conclusion, small molecules and biotechnological drugs have opened a new frontier for scientific research and offer hope for patients who, until not more than ten years ago, lacked effective therapy for a disabling, distressing, and alienating disease such as AD.

## Figures and Tables

**Table 1:** Overall population: dermatological symptoms (itching NRS and sleep NRS) and signs (EASI score) at baseline, visit 1 (3 months from treatment starting) and visit 2 (6 months from treatment starting). ULN: upper limit of normal. AD clinical phenotype: 1—classic; 2—head and neck eczema; 3—portraits of dermatitis; 4—generalized eczema.

	Sex	Age	Age of Onset AD	Duration of AD	AD Clinical Phenotype	Head and Neck Involvement	Duration of Treatment with Dupilumab (Months)	Elevated IgE (>ULN)	EASI Baseline Dupilumab	EASI Baseline Upadacitinib	Itch-NRS Baseline-Upadacitinib	Sleep-NRS Baseline-Upadacitinib	EASI W12 Upadacitinib	Itch-NRS W12 Upadacitinib	Sleep-NRS W12 Upadacitinib	EASI W24 Upadacitinib	Itch-NRS W24 Upadacitinib	Sleep-NRS W24 Upadacitinib
**Patient 1**	M	25	5	20	2	Yes	9	No	30	15	7	10	0	0	0	0	0	0
**Patient 2**	F	54	1	53	3	Yes	39	No	25	16	8	0	0	0	0	0	0	0
**Patient 3**	M	24	16	9	4	Yes	36	Yes	40	15	10	4	4	4	0	0	2	0
**Patient 4**	M	19	15	3	1	Yes	29	Yes	25	15	10	10	0	4	0	3	5	0
**Patient 5**	F	43	0	43	4	Yes	6	No	40	30	10	10	8	8	7	5	3	1
**Patient 6**	M	42	19	23	1	Yes	29	Yes	52	30	8	7	0	0	0	2	3	0

**Table 2:** Ocular signs (mucous fishing, corneal neovascularization, conjunctival hyperemia, papillary reaction, tarsal fibrosis, BUT, NEI score, corneal opacities) at baseline (W0), visit 1 (W12), and 2 (W24). Mucous fishing (0–1), corneal neovascularization (0–3), conjunctival hyperemia (0–4), papillary reaction (0–3), tarsal fibrosis (0–1), BUT (normal values >10 s), NEI score (0–15), corneal opacities (0–3). MF: mucous fishing. CNV: corneal neovascularization. CH: conjunctival hyperemia. TP: tarsal papillae. TF: tarsal fibrosis. BUT: tear break-up time. NEI: National Eye Institute corneal fluorescein staining. CO: corneal opacities.

	W0																W12														W24																	
	MF		CNV		CH		TP		TF		BUT		NEI		CO		MF		CNV		CH		TP		TF		BUT		NEI		CO		MF		CNV		CH		TP		TF		BUT		NEI		CO	
	RE	LE	RE	LE	RE	LE	RE	LE	RE	LE	RE	LE	RE	LE	RE	LE	RE	LE	RE	LE	RE	LE	RE	LE	RE	LE	RE	LE	RE	LE	RE	LE	RE	LE	RE	LE	RE	LE	RE	LE	RE	LE	RE	LE	RE	LE	RE	LE
**Patient 1**	1	0	0	0	2	2	2	2	1	1	5	6	0	1	0	0	1	0	0	0	0	0	1	1	1	1	6	5	0	0	0	0	0	0	0	0	0	0	1	1	1	1	4	3	0	0	0	0
**Patient 2**	0	1	0	0	2	2	2	2	1	1	2	1	0	2	0	0	0	0	0	0	0	0	1	1	1	1	3	2	0	0	0	0	0	0	0	0	0	0	1	1	1	1	5	3	0	0	0	0
**Patient 3**	1	1	1	1	4	4	2	2	1	1	12	11	0	0	0	0	0	0	1	1	0	1	1	1	1	1	13	12	0	0	0	0	0	0	0	0	0	0	1	1	1	1	10	8	0	0	0	0
**Patient 4**	1	1	1	1	4	4	3	3	0	0	2	4	3	1	0	0	0	0	0	0	0	1	2	2	0	0	4	5	0	0	0	0	0	0	1	1	0	1	1	1	0	0	7	8	1	0	0	0
**Patient 5**	0	0	0	0	0	0	1	1	1	1	4	2	0	0	0	0	0	0	0	0	0	0	1	1	1	1	5	4	0	0	0	0	0	0	0	0	0	0	1	1	1	1	6	6	0	0	0	0
**Patient 6**	1	1	3	2	3	3	1	1	1	1	3	4	0	0	2	1	0	0	2	2	0	0	1	1	1	1	5	6	0	0	2	1	0	0	1	1	0	0	1	1	1	1	6	7	0	0	2	1

## Data Availability

The original contributions presented in the study are included in the article, further inquiries can be directed to the corresponding author.
